# Refers to: Association of Nocturnal Enuresis With Vesico-ureteral Reflux and Renal Cortical Damage

**DOI:** 10.5812/numonthly.6888

**Published:** 2012-09-24

**Authors:** Kristian Vinter Juul

**Affiliations:** 1 Ferring Clinical R & D, Medical Science Urology, Copenhagen S, Denmark; 2 University of Ghent, Ghent, Belgium

**Keywords:** Nocturnal Enuresis, Vesico-ureteral Reflux

*Dear Editor*,

In the mid-1980’s, the Aarhus group headed by the two pioneers in Nocturnal Enuresis research Jens-Christian Djurhuus and Jens Peter Nørgaard, reported the first data correlating the degree of Vesicoureteral Reflux (VUR) and associated nephropathy to the presence of enuresis (1). The material showed that one half of the patients with reflux also had enuresis, and that in spite of identical demographics and medical history, patients with enuresis presented a significantly lower degree of reflux nephropathy, leading to the hypothesis that enuresis might act as a safety valve mechanism reducing the possibility of VUR nephropathy.

The new data presented by Dr. Naseri suggest that VUR is uncommon in children with monosymptomatic nocturnal enuresis (MNE) and in daytime continent non-monosymptomatic nocturnal enuresis (NMNE) while being relatively more common in enuretic children who have daytime incontinence. In contrast to previous studies, no correlation between VUR and urinary tract infection (UTI), secondary enuresis, or any other daytime symptoms except for incontinence was found. This finding warrants further research.

Also, the finding of (a) gender difference in VUR being significantly more frequent in enuretic girls raises important clinical questions. Is the apparent association between gender and VUR in enuretic children, if proved in future research, acquired or genetic? Some studies have suggested the presence of a modified VUR gene (s) on the X-chromosome, accounting for the higher incidence of this disorder among female members, while on the other hand male-to-male transmission and a higher ratio of females to males has provided arguments against X-linked inheritance (2). If X-linked inheritance of VUR is the case, this will be an intriguing exception to the general finding that in genetic kidney diseases males are often more vulnerable to mutations in their single copy of X-linked genes, whereas females having a mixture of cells expressing different sets of X-linked genes are often protected from severe clinical manifestations due to X-linked mutations (3). For future genetic research it will be interesting to explore if a genetically distinct subgroup of VUR with severe reflux exist with female preponderance.
